# Review of Factors Affecting the Growth and Survival of Follicular Grafts

**DOI:** 10.4103/0974-2077.69014

**Published:** 2010

**Authors:** William M Parsley, David Perez-Meza

**Affiliations:** *Department of Dermatology, University of Louisville Medical Center, Louisville, Kentucky, USA*; 1 *Plastic Surgery and Hair Transplant Surgery, Mexico City, Mexico, USA*

**Keywords:** Hair transplantation, storage solutions, ischemia reperfusion injury, platelet-rich plasma

## Abstract

Great strides have been made in hair restoration over the past 20 years. A better understanding of natural balding and non-balding patterns along with more respect for ageing has helped guide proper hairline design. Additionally, the use of smaller grafts has created a significantly improved natural appearance to the transplanted grafts. Inconsistent growth and survival of follicular grafts, however, has continued to be a problem that has perplexed hair restoration surgeons. This review attempts to explore the stresses affecting grafts during transplantation and some of the complexities involved in graft growth and survival. These authors reviewed the literature to determine the primary scope of aspects influencing growth and survival of follicular grafts. This scope includes patient selection, operating techniques, graft care, storage solutions and additives. The primary focus of the hair restoration surgeons should first be attention to the fundamentals of hair care, hydration, temperature, time out of body and gentle handling. Factors such as advanced storage solutions and additives can be helpful once the fundamentals have been addressed.

## INTRODUCTION

In hair transplant surgery (HTS), identifying and modifying the factors that affect graft survival have received a great deal of attention. Important factors include the patient’s health, hair characteristics and the operative techniques. With reported survival rates of 90% to well over 100%,[1] it is reasonable to question the relative benefit of efforts to increase graft survival. The problem is that studies are generally performed in ideal circumstances with vigorous terminal hair and small isolated study boxes. The reality is that few doctors reach 100% survival in common practice when the totality of the grafts is measured. In less-than-ideal situations, graft survival may drop well below 100% by the estimate of many experienced hair surgeons. This article intends to give a brief synopsis of some of the major issues involved while emphasizing the more recent studies.

The factors affecting graft survival are discussed under the following headings:

1. Donor scalp issues
2. Operation day factors, which include physical damage to the grafts-dehydration, transection, blunt trauma, ischaemia-reperfusion injury (IRI) and storage solutions

## PATIENT SELECTION: DONOR SCALP ISSUES

To understand the issues, it needs to be emphasized that hair follicles exit the scalp singly (about 10% or more) or in groups of two to five follicles. These groups were described in 1984 as most commonly containing one to four follicles per group by Headington,[2] who termed them ″follicular units″. With androgenetic alopecia (AGA), individual follicles ″miniaturize″ and either disappear or become vellus-like hairs. Therefore, the number of follicular units and the number of follicles in the follicular units decrease with time. While the follicular units in the optimal donor area of the occipital and parietal scalp are ″relatively″ protected from androgenetic hair loss, even those follicular units may be somewhat affected with time. Robust terminal hair follicles and hair follicles in groups have a better HTS survival than weaker hairs and single hair follicular units. Their vigor can be improved slightly with finasteride and minoxidil,[3–5] but it is not likely that the patient will continue indefinitely with medical treatment. Additionally, miniaturizing hair and smaller follicular units usually mean that the donor zone is being affected by AGA and that the transplanted grafts can be expected to considerably weaken with time. For these reasons, patient evaluation is an important and often underrated step before considering surgery.

Therefore, not all patients are good candidates for hair restoration. As mentioned, fine, sparse, weak donor hair often results in poor coverage and poor survival. If the patient is young and significant further hair loss is anticipated, the problem is magnified. Poor hair characteristics also present challenges in hairline design. Aggressive hairline designs may not withstand the ravages of continued loss. On the other hand, it also needs to be emphasized that while these patients may not have dramatic results, they can often be cosmetically ″improved″ and be pleased with the results. If expectations are realistic and future loss is carefully considered, then a successful outcome is often achievable in borderline patients. Cases where modest improvement is the best that can be anticipated, require the greatest skill of the doctor and the assistants; from hairline design to gentle care for the grafts in preparation and in placing into the recipient site.

## OPERATIVE DAY FACTORS

The day of surgery is the most important single event in the control of the medical team. Harvested hair grafts take approximately 3 days before starting to regain their blood supply.[6,7] During the intra-operative and post-operative periods, the graft is subject to many stresses, any of which can result in compromise or death of the hair graft. Among these are dehydration, mechanical trauma, hypoxia, ATP depletion, ischaemia reperfusion injury, cold injury and lactic acid accumulation. Work to find better storage solutions and additives has received special attention.

### Physical damage to the grafts: Dehydration, transection, blunt trauma

Loss of grafts to dehydration has been documented in several studies, but reported survival time in a dry environment (glove, Telfa pad) is quite variable, from 3 min to greater than 16 min, before significant graft death occurs. Transection of grafts during harvesting and preparation results in loss of survival and finer, weaker hairs on regrowth, but these studies also show great variation in results. In addition, blunt trauma to the bulb region and to the bulge zone results in loss of survival, with trauma to the bulge zone being more damaging. Interestingly, there is suggestive evidence that chilling of the grafts offers some protection from blunt trauma to the bulge.[8–10]

## ISCHAEMIA–REPERFUSION INJURY

During transplantation, hair tissue is separated from its blood supply and develops ischaemia. In hypoxic conditions, ATP is broken down but the degradation stops at hypoxanthine and xanthine because the conversion to uric acid requires xanthine oxidase, which cannot function without oxygen. This creates a build up of hypoxanthine and xanthine in the cells. In organs susceptible to ischemic reperfusion injury (IRI), upon reperfusion and exposure to oxygen, the sudden conversion of hypoxanthine and xanthine via xanthine oxidase to uric acid creates free radicals and reactive oxygen species,[11] thereby overwhelming the body’s antioxidant defense and starting a cascade often leading to aponecrosis [apoptotic cell death (ACD) and necrosis]. These free radicals released are particularly damaging to the double strands of DNA and also to the cell membrane, where they cause lipid peroxidation. Lipid peroxidation of the cell membrane releases malondialdehyde (MDA) and 4-hydroxyalkenals (HAE), which can be used as measurements of free radical activity. DNA breakdown during ACD can be measured by cytoplasmic histone-associated DNA fragments (HADF).

Most transplanted organs are surgically reconnected to the body’s blood supply and are exposed to a sudden dramatic rise in oxygen tension. In contrast to large organ transplants, hair grafts are perfused passively for atleast 3 days before being revascularized thus not receiving such a sudden ″blast″ of oxygen. For this reason, some question whether or not IRI occurs in hair transplantation. Cooley[12] used a standard test for free radicals, an MDA assay, to test 150 grafts in seven patients. The test grafts were placed into the scalp and later removed to complete the iscahemia/reperfusion cycle and then tested against control grafts that were never reimplanted. The MDA assay in test grafts revealed that MDA levels elevated 200–600% over controls. Krugluger *et al*.[13] demonstrated a dramatic rise in cytoplasmic histone-associated DNA fragments (cHADF) after 36 h of culture in serum-containing DMEM culture media. In addition, cHADF was significantly reduced by storage in media containing antioxidants. In yet another study, Krugluger reported better growth and less shedding after adding various antioxidants to holding solutions.[14] Thus, free radical damage to both the cell membrane and the DNA has been demonstrated in these studies to occur in hair grafts, with the cell membrane damage shown to increase with reperfusion. While more studies are needed, there certainly appears to be suggestive evidence for the existence of IRI and related ACD in hair grafts. This also gives more validity to the use of antioxidants in storage solutions.

### Storage solutions

Different considerations need to be taken into account about storage solutions. These include ionic concentrations, pH, osmolality, and additive substances.

#### Extracellular versus intracellular balanced solutions

Extracellular fluid has a high Na^+^ and a low K^+^ concentration, while intracellular fluids have the opposite (low Na^+^ and high K^+^). With cold storage, the Na^+^/K^+^ pumps and Ca^2+^ channels are shut down, with the potential to create an ionic imbalance. Normal saline, lactated Ringer’s, tissue culture media and plasma-lyte A have an extracellular ionic balance, which could make grafts in chilled storage more susceptible to electrolyte imbalance and cell swelling as sodium rushes into the cells. Intracellular-type preservation solutions (HypoThermosol, Viaspan, Custodial) have a lower Na^+^ concentration and select additives, but are more expensive than extracellular balanced solutions [Table 1]. The small number of reports published so far seem to suggest at least a modest benefit if these advanced solutions are used within the normal surgical time durations of 6–8 h when compared to normal saline, but greater benefit if graft insertion is delayed beyond this duration.[15]

**Table 1:** Storage solutions

Medium	pH	Osmolality	Buffer	Comments
Human serum	7.4	289 (280–310)	Bicarbonate	Bicarbonate is a suboptimal pH buffer for cold storage solutions
Normal saline	5.0	308	None	Extracellular balance
Lactated Ringer’s	6.5	273	Lactate	Extracellular balance
Plasma-lyte A	7.4	294	Acetate gluconate	Extracellular balance
HypoThermosol-FRS	7.6	360	HEPES	Intracellular-like balance
HTK-Custodial	7.4–40C 7.25–RT	310	Histidine	Intracellular-like balance Tryptophan (membrane stabilizer) Ketoglutarate (energy substrate)
Sterile water	5.5	0	None	Mistakenly using this as a storage solution can kill all grafts

#### pH

Unbuffered normal saline (UNS) is designed for intravenous before (IV) use and has a variable pH, usually in the range of 5.0. Normal human serum has a pH of 7.4. Increasing acidity has a known negative effect on tissue survival, but the effect on follicular tissue pH when using UNS is not known at this time. Researchers generally buffer normal saline with phosphate (PBS) before conducting tissue studies. Plasma-lyte A has a pH of 7.4, using an acetate buffer. DMEM most commonly contains a bicarbonate buffer and is designed to be used at 37 degrees *in vitro* in controlled chambers with 5–10% CO_2_, which allows the bicarbonate buffer to work properly. In open air, a bicarbonate buffer can become alkaline and may not be healthy for hair grafts. The more expensive HEPES buffer has been used with sterile DMEM solution and has been used in few *in vivo* hair studies. But, it should be noted that DMEM is not specifically approved as a transplant storage media (personal correspondence with Sigma-Aldrich Co.) and its sterile solution is not available, unless specifically requested. Advanced intracellular balanced solutions most commonly use HEPES, particularly in those meant to be chilled, as it adapts to temperature changes. For chilled storage solutions, a pH of slightly above 7.4 is ideal.

#### Osmolality

Osmolality of normal serum ranges from 280 to 310 mOsmol/L. UNS has an acceptable osmolality of 308. Advanced solutions use osmotic stabilizers because the body naturally has a higher concentration of impermeable solutes intracellularly than extracellularly. Membrane pumps are altered during cold storage. Adding impermeable solutes such as lactobionate, dextran and mannitol as osmotic stabilizers to the storage solution helps to maintain the proper osmotic balance, particularly in chilled solutions, thus reducing cell swelling. These complex sugars provide very little energy but work well for osmotic control.[16]

#### Additives

An area of considerable interest has been the use of storage solution additives to enhance growth and survival of hair grafts. Among the additives that have shown positive effects are allopurinol, nitric oxide inhibitors, arachidonic acid inhibitors, vitamins, [Table 2] adenosine triphosphate, insulin, mannitol, amino acids and steroids. Most of the studies have been *in vitro* studies (outer root sheath cell culture, hair shaft elongation ″(HSE) in culture and implantation into nude mice), but some have been *in vivo*. Multiple studies indicate that the storage medium and additives during the first 5 h after donor harvesting can have an effect on the length of survival. Long-term storage indicates an even greater importance of the media type [Table 3]. Both *in vitro* and *in vivo* studies have shown the beneficial role of these additives on HSE.

**Table 2:** Effect of addition of vitamin B12 on hair shaft elongation

%HSE in DMEM	%HSE in PBS without vit B12	%HSE in OBS with 2.5 *µ*g/mL vit B12	%HSE with 25 *µ*g/mL vit B12
17.6	1.2	22	27

HSE- hair shaft elongation

**Table 3:** Holding solution and additives

Additive	Type of study	Incubation time	Result
ATP-MgCl (energy source)	*In vitro*	5 h in UNS at 26°C	↑Survival in culture at 10 days
Aminoguanidine (inhibitor of iNOS)	*In vitro*	5 h in DMEM with HEPES at 26°C	↑HSE ↓cHADF
14,15 epoxyeicosatrienoic acid (inhibits arachidonic acid)	*In vitro*	5 h in DMEM with HEPES at 26°C	↑HSE ↓cHADF
Vitamin B12 (inducer of cell proliferation)	*In vitro*	5 h in PBS at 26°C	↑HSE Induction of β-catenin transcription
Aminoguanidine (inhibitor of iNOS)	*In vitro*	3 h in TCM at 26°C	No post-operative shedding (0/6)
PRP (contains growth factors)	*In vitro*	15 min	15% ↑ in graft survival

#### In vitro studies: ATP-MgCl with deferoxamine

Raposio *et al*. reported that enhancing normal saline with ATP-MgCl and deferoxamine showed improved graft survival.[17] ATP-MgCl was used as an energy source. Deferoxamine, a potent non-selective free radical scavenger, was used as a chelating agent for iron, thus reducing the iron-mediated conversion of hydrogen peroxide into the potent hydroxyl radical. Normal saline (control) was compared to the ″enhanced″ saline by storing grafts in these solutions at room temperature (RT) for 5 h. The grafts in the control and experimental groups were then placed in Williams E medium and cultured in a controlled CO_2_ chamber for 10 days. Survival was determined by the preservation of follicular architectural features. The grafts in the enhanced solution had a 98% survival rate compared to 87% for the control.

Recent unpublished work with a liposomal formulation of ATP has shown potential for improving graft survival for extended *ex vivo* storage periods, (Personal communication with Dr. Jerry Cooley).

#### Aminoguanidine (AMG, nitric oxide inhibitor)

Krugluger[18] found that grafts stored for 5 h in DMEM with AMG at 10 µg/mL resulted in a significant increase in HSE. AMG added to PBS showed no effect. Also, there was a significant reduction in cHADF, a measure of ACD, after culture for 36 h. No increase in cytoplasmic HAD was noted in any grafts until 36 h. A later study[19] showed a 14-fold induction of vascular endothelial growth factor (VEGF) mRNA after 5 h culture of outer root sheath cells (ORS) in PBS enhanced with AMG. AMG at 100 µg/mL gave the best result. No increase in VEGF mRNA was noted in cultured dermal papillae (DP) cells, backing up Yano’s earlier study indicating that ORS cells produce VEGF not DP cells, as previously thought.

#### 4,15-epoxyeicosatrienoic acid (EET)

In Krugluger’s study (above), EET was also shown to have a significant effect on HSE.

#### Vitamin B12

Vitamin B12 added to storage solutions for 5 h followed by organ culture for 3 days,[20] Vitamin B12 allowed a dose-dependent hair shaft elongation increase as follows:

Real-time polymerase chain reaction showed induction of β-catenin transcription (a key molecule in the wnt pathway) by analysis of mRNA transcription of intracellular hair follicle growth-promoting molecules.

#### In vivo studies: AMG

Krugluger *et al*.[14] found that DMEM containing inhibitors of inducible nitric oxide synthase (iNOS) prevented post-transplant hair shedding of grafts in six of six patients. The primary inhibitor of iNOS was AMG. Nitric oxide is an important regulator in wound healing, but can be damaging to tissue in excess amounts. A primary mechanism of damage is the interaction of the superoxide ion and nitric oxide to form peroxynitrite, which is highly damaging to the DNA and cell membranes. In the same study, DMEM containing arachidonic acid inhibitors prevented graft hair shedding in five of six patients vs. 0 of six in controls. Both additives also demonstrated significant improvement in hair shaft elongation studies. Positive results, both *in vitro* and *in vivo*, make AMG and arachidonic acid inhibitors additives to watch closely as more studies are performed.

#### Platelet-rich plasma

Wound healing occurs as a sequential cascade of overlapping processes and requires the coordinated completion of a variety of cellular activities. Each step during the process is orchestrated by varying levels of many growth factors and by differential expression of their receptors. Growth factors are the engines, or modulating factors, that drive wound healing.[21–24] Beginning in the 90s, polypeptide growth factors have emerged as the ″Holy Grail″ in wound repair. Platelets release large amounts of platelet-derived growth factor (PDGFaa, PDGFbb and PDGFab), transforming growth factor beta (TGFβ1 and β2), epidermal growth factor (EGF) and VEGF. The circulating platelet participates in natural wound healing based on its number in circulating blood. It further enhances wound healing by virtue of its concentration as Platelet-rich plasma (PRP).

PRP is an autologous concentration of human platelets in a small volume of plasma that has a higher platelet concentration (4–7-times) above baseline. PRP is obtained from the patient’s own blood after processing in an automated centrifuge.[25] Manual centrifuges are not recommended, not only due to their potential for product contamination but also due to their decreased efficiency in platelet recovery (30–70%) as compared to automated devices.[26] In 2007, the FDA approved the use of automated centrifuges for the preparation of growth factors to be used in chronic ulcers of diabetic patients.

There are also several reports demonstrating that the content of growth factors in PRP can vary tremendously, depending on the automated system used. Variations in key properties of the PRP, including platelet concentration, type of clot activator, using or not using a clot activator, etc., may markedly influence the different biological effects.

PRP has been used in the past in plastic surgery, dental surgery, general surgery, neurosurgery, orthopedic surgery, etc.[27–29] to reduce bleeding, swelling, prevent infection and to speed up the wound-healing process. The application of PRP in surgery has produced conflicting results – positive in some publications[30,31] and negative results in others.[32,33] It appears that PRP probably has a greater effect in chronic wounds, compromised wounds, poor vascularized tissue or infection.

Platelet-poor plasma, which is obtained during the separation and concentration process, does not contain growth factors and can be used only as a sealant or haemostatic agent (fibrin glue).

Perez-Meza *et al*., in part 1 of their growth factors study,[7,34] found that growth factors appear to play a key role in the wound-healing process and revascularization of the hair graft following HTS. There are only two hair growth and survival studies using PRP in HTS. In the first one, Perez-Meza *et al*.[6] studied, used automated centrifuges and applied PRP in the donor and recipient area, including a graft storage study. They included 10 patients for the wound-healing study and three of them for the hair-survival study. The graft-survival study areas were selected; three boxes (1 cm^2^ each) were marked in each side of the scalp for the PRP study and the placebo control study, respectively. Twenty sites were made using a 1.3 Minde blade, 4 mm deep. Two hair FUs were placed. The grafts of the PRP group were preserved in the PRP solution (non-activated) and soaked in PRP gel 10’ (activated) before placing. At 1-year follow-up, hair counts were similar between the two groups. At the ISHRS 2005 meeting, Sydney, Australia, Perez-Meza D presented the results.[36]

In 2005, Uebel performed the second study using a manual centrifuge. He included 23 patients. Two areas (2.5 cm^2^) each were marked in the scalp and planted at 20 g/cm^2^. At 1-year, the area treated with PRP demonstrated a yield of 18.7 FU/cm^2^ vs. 16.4 FU/cm^2^ of the placebo group, an increase in follicular density of 15.1%.[37]. Further controlled studies including more patients and automated devices are needed.

## EFFECT OF CHILLING

A basic question of principle must be answered with the storage solution and its temperature. Is it better to try to reduce the metabolic activity of the tissue to be grafted or to keep the graft metabolically active? If the tissue is chilled, then cold injury must be addressed. If the tissue is to be kept active, then oxygen and energy requirements must be considered along with the elimination of by-products and increasing tissue acidity.

### Chilling vs. room temperature

The two primary studies comparing chilled and room temperature (RT) storage of hair grafts are *in vitro* studies. Raposio *et al*. studied chilled (1°C) vs. RT grafts stored in normal saline. There was no significant difference in survival of the grafts after 5 h of storage followed by hair shaft elongation studies.[38] Hwang performed a similar but larger study, storing the chilled and RT grafts for 6, 24 and 48 h before hair shaft elongation evaluation. Survival was similar through 6 h but dropped off dramatically at 24 and 48 h.[39]

Several factors must be considered in deciding between chilled and RT graft storage. Large organs can survive up to 12 times longer in chilled storage compared to RT storage.[40] The exact duration of RT storage, where the significant drop in hair graft survival occurs, is not known, other than the fact that it is between 6 and 24 h. The lack of *in vivo* studies also leaves some questions unanswered. Because the planting phase is by-passed, we do not know the affect that insertion trauma may have on chilled grafts compared to RT grafts. Beehner’s study indicated that chilled grafts withstood a hard crush to the bulge better than RT grafts (38% compared to 0% survival).[41] Also, there are better storage solutions available to protect against cold injury than normal saline. Further research in this area is much needed.

### Freezing for long-term storage

In 2002, Adanali *et al*. reported that grafts frozen for 2 weeks at –20°C (standard freezer) showed no damage under light microscopic examination, suggesting that this might allow long-term graft preservation.[42] Later, Jimenez performed a study of 150 grafts frozen for 1 h, 5 days and 7 days at –20°C before implantation.[43] Survival after freezing for 1 h was 20%; 5 days, 0%; 7 days, 0%. This demonstrates the unreliability of light microscopy (LM)to evaluate survival. At –20°C, ice crystals are constantly forming and reforming, killing the cells. Freezing tissue for storage requires much colder temperatures in order to create a ″glass formation state″ (no crystal movement), usually with liquid nitrogen. This process involves using cryoprotectants in which modifications for tissue type and timing of the freeze/thaw are critical. Graft survival of 50% has been demonstrated in nude mice, at 8 months after transplantation, using grafts which were frozen for a month in liquid nitrogen.[41]

### Time out of body

In 1974, Unger performed an *in vivo* study with 4 mm grafts in comparing survival after the following intervals from graft harvesting to graft placement: 2 min, 30 min and 60 min.[44] Surprisingly, survival at 2 min time out of body (TOB)was 84% while survival at 30 min was 98% and at 60 min was 97%. Storage was in chilled NS. Limmer performed an *in vivo* TOB study using chilled NS with follicular unit grafts. The results were: 2 h, 95%; 4 h, 90%; 6 h, 86%; 8 h, 88%; 24 h, 79%; 48 h, 54%.[45] Limmer related a good ″rule of thumb″, stating that the loss was roughly 1%/hour. Better storage solutions may improve these statistics.

### Effect of density on survival

One of the original density studies was carried out by Mayer *et al*., using an 18G needle for planting 2-hair FUs at four different densities.[46] The results were 10/cm^2^, 97%, 20/cm^2^, 92%, 30/cm^2^, 72%, 40/cm^2^, 78%. Beehner followed this with a two-patient study of graft survival at different densities using smaller needles (19 and 20G).[47] There was considerable variation in graft survival, but the smaller sites appeared to have an advantage. Subsequently, Nakatsui had graft survival of around 98% at densities of 61 and 72%, with 0.8–0.9g custom blades,[48] and Tsilosani[49] reported two patients in whom densities of 100 FU/cm^2^ were planted into sagittal 1.0 mm slits. It certainly appears that survival at higher densities is achievable, but questions remain as to whether or not it is consistently reproducible, particularly in patients with weaker grafts, with health issues or with a compromised recipient site

### Supplemental oxygen

The most critical stress on a graft is the sudden separation from its oxygen supply and its associated depletion of ATP. Thus, it is not surprising that efforts have been made to use supplemental oxygen in hair restoration surgery. Unfortunately, there are only sporadic anecdotal reports and no conclusions are yet possible. So what are the established pertinent effects of supplemental oxygen on the skin and scalp? Eighty percent inhaled oxygen can reduce the post-operative infection rate by 50%. Any change in oxygen tension up-regulates VEGF, but angiogenesis can only occur with adequate oxygen tension.[50] Oxygen can be supplemented in several ways: general hyperbaric oxygen, local hyperbaric oxygen, inhaled normobaric oxygen, local normobaric oxygen and topical oxygen emulsion. Most of its use has been on wound healing, but there have been some unpublished anecdotal reports of improved hair graft survival with post-operative hyperbaric oxygen in an oxygen chamber. A potential problem is that oxygen can also create ″oxidative stress″. This can certainly occur with generalized hyperbaric oxygen; however, inhaled normobaric oxygen is unlikely to cause oxidative stress for at least 12 h. More studies are needed on the use of oxygen in hair restoration. At this time, any benefits to graft survival are unproven.

## SUMMARY

Many factors influence graft survival, and it is often difficult to determine specifically their relative importance. Certainly, it is helpful to review studies on other transplanted tissues and try to determine those factors that are universal. Finding the optimal storage solution and the most effective additives appear to be a worthwhile pursuit, when there is confidence that the basics of graft care have been addressed. If performed improperly, several areas of the HTS procedure can each result in graft death; but, it is likely that the grafts accumulate sublethal stresses from each stage of the procedure, which, in their totality, can lead to loss of survival. Addressing all issues is of paramount importance to ensure better graft survival.
